# Bleach baths enhance skin barrier, reduce itch but do not normalize skin dysbiosis in atopic dermatitis

**DOI:** 10.1007/s00403-023-02723-1

**Published:** 2023-09-27

**Authors:** Ania Stolarczyk, Nelissa Perez-Nazario, Sara A. Knowlden, Ellen Chinchilli, Alex Grier, Amy Paller, Steven R. Gill, Anna De Benedetto, Takeshi Yoshida, Lisa A. Beck

**Affiliations:** 1 Department of Dermatology, University of Rochester Medical Center, 601 Elmwood Ave, Box 697, Rochester, NY 14642 USA; 2 University of Rochester Genomics Research Center, University of Rochester Medical Center, Rochester, NY USA; 3 Department of Microbiology and Immunology, University of Rochester Medical Center, Rochester, NY USA; 4 Department of Dermatology, Northwestern University, Chicago, IL USA

**Keywords:** Atopic dermatitis, Bleach baths, *Staphylococcus aureus*, Skin barrier, Itch, Skin microbiome

## Abstract

Studies have demonstrated that bleach baths improve atopic dermatitis (AD) severity; however, the effects on itch, skin barrier, and cutaneous microbial composition are less clear. We examined whether bleach baths reduce itch, normalize skin barrier function, reduce *S. aureus* absolute abundance, and increase microbial diversity in adults with AD who were colonized with *S. aureus* on their non-lesional skin. This was an open label, non-randomized, controlled trial performed at a single academic center. Fifteen AD and five non-atopic healthy controls (NA) were instructed to take two bleach baths (0.005% NaClO; 5–10 min duration) per week for a total of 12 weeks as add-on therapy. Adults 18 to 65 years (inclusive) with mild to severe AD were recruited with EASI score > 6.0, *S. aureus* culture positivity, access to a bathtub, and ability and willingness to maintain current topical or systemic treatments. They were evaluated at baseline (before bleach baths), 6 weeks, and 12 weeks after the intervention of twice-weekly bleach baths. Efficacy measurements included EASI as well as 5-D Pruritus and ItchyQoL™. Transepidermal water loss (TEWL) and stratum corneum (SC) integrity assay were performed to assess the skin barrier. Skin dysbiosis was measured by *S. aureus* cultivation, *S. aureus* abundance (qPCR of thermonuclease gene), and V1-V3 16S rRNA gene sequencing on non-lesional and lesional AD skin. After 12 weeks of bleach baths, 8/15 (53.3%) AD subjects achieved an EASI_50_ and a significant reduction in itch as measured by 5-D pruritus and Itchy QoL. Eighty-seven percent reported improvements in sleep quality. At study entry, AD subjects had higher non-lesional TEWL values than NA subjects, and only AD subjects experienced a reduction with bleach baths (*p* = 0.006). Similarly, SC integrity improved as early as 6 weeks after bleach baths in AD subjects. Notably, bleach baths had no significant effect on *S. aureus* culture-positivity, qPCR absolute abundance, or microbial diversity. The addition of twice-weekly bleach baths improves investigator-assessed AD severity, patient-reported pruritus and sleep as well as physiological measures of skin barrier function in adult AD subjects while having no effect on qualitative and quantitative measures of cutaneous *S. aureus*. *Trial Registration*: ClinicalTrials.gov Identifier: NCT01996150, Date of registration: November 27th, 2013.

**Supplementary Information:**

The online version contains supplementary material available at 10.1007/s00403-023-02723-1.

## Introduction

Atopic dermatitis (AD) is a common inflammatory skin disease, affecting 15–20% of children and 1–3% of adults worldwide [1]. It is characterized by skin barrier disruption, type 2 immunity, and reduced quality-of-life (QoL) resulting from the itch-scratch cycle. AD patients also exhibit skin bacterial dysbiosis with high rates of *Staphylococcus aureus* (*S. aureus*) colonization especially in subjects with more severe disease and greater type 2 polarization [2–6]. Whether disease severity begets *S. aureus* colonization or chronic colonization leads to the worsening of the disease is not clear. Safe and cost-effective topical therapies for the management of AD are still limited and whether therapies that primarily act by normalizing skin dysbiosis are effective has not been adequately tested.

*Staphylococcus aureus* is not part of the normal human skin flora, but can be cultured from lesional skin in up to 100% of AD subjects and is strongly linked to AD flares and severity [2, 5, 7–9]. This *S. aureus* colonization is thought to be due to decreased levels of antimicrobial peptides and the expression of *S. aureus* adhesins [10, 11]. Virulence factors released by *S. aureus* are thought to play a role in skin barrier disruption and their effects are enhanced in the presence of type 2 cytokines [12, 13]. Current guidelines support the use of antistaphylococcal antibiotics to treat overt skin infections but are not recommended to treat the staphylococcal colonization commonly seen in AD subjects [14].

Several topical antistaphylococcal approaches, including antibacterial soaps, antibacterial bath additives, and bleach baths have been claimed to improve AD severity and/or decrease secondary infections, but only bleach baths have become “standard of care” as an add-on therapy [15, 16]. This treatment first gained attention when a 2009 placebo-controlled study found that children with *S. aureus*-infected, moderate-severe AD exhibited significant reductions in disease severity as measured by the Eczema Area and Severity Index (EASI) after an oral antibiotic course and 3 months of maintenance therapy with twice-weekly bleach baths (0.005% sodium hypochlorite [NaClO]) used in conjunction with monthly intranasal mupirocin [17]. Since then, several studies have also observed that bleach baths improve disease severity [18–22]. The 2009 study found that contact with the bleach-containing water was important, as the best improvements were seen with submerged body parts. However, no change in *S. aureus*-positivity was observed [17].

The mechanism of action for bleach baths in AD remains unclear. Studies have shown that a much higher concentration of NaClO (0.04%) is needed to reduce *S. aureus* derived from AD skin ex vivo [23]. One study found no bacteriocidal effect of NaClO on *S. aureus* (or *S. epidermidis*) growth (log phase or stationary) until > 0.03% concentration [24]. This group further modeled the effect of 0.005% NAClO on *S. aureus* CFUs in porcine skin, where they also found no bactericidal effect [24]. Further study is required to better understand the effects of bleach baths on the entire skin microbial community. It is possible that bleach baths may have an anti-inflammatory effect, as NaClO has been shown to decrease the expression of NF-κB associated genes in human keratinocytes [25].

In addition, there is limited data on the effects of bleach baths on barrier function in AD. Shi et al. conducted a split-body, randomized, controlled trial and found that bleach baths do not significantly affect transepidermal water loss (TEWL), stratum corneum (SC) hydration, or pH compared to tap water baths when assessed at baseline and up to 60 min post bleach bath immersion [26]. Further, a randomized, placebo-controlled crossover trial of 40 pediatric subjects with AD found no significant difference in TEWL or SC hydration after 4 weeks of at least twice-weekly bleach bath treatment versus tap water, however, this study had a high non-adherence rate (*n* = 14) [27]. Little is known about how barrier function is affected in the presence of a longer bleach bath intervention period.

To assess whether bleach baths normalize skin barrier function and dysbiosis and/or reduce itch, we performed an open-label trial in adult AD subjects colonized with *S. aureus*. These parameters were studied at baseline and after 6 and 12 weeks of 0.005% sodium hypochlorite baths twice-weekly. We compared these observations with what was observed in a cohort of non-atopic (NA) healthy adult controls, treated in parallel with the same bleach bath regimen.

## Results

### Bleach baths reduce atopic dermatitis disease severity

**Fig. 1:**
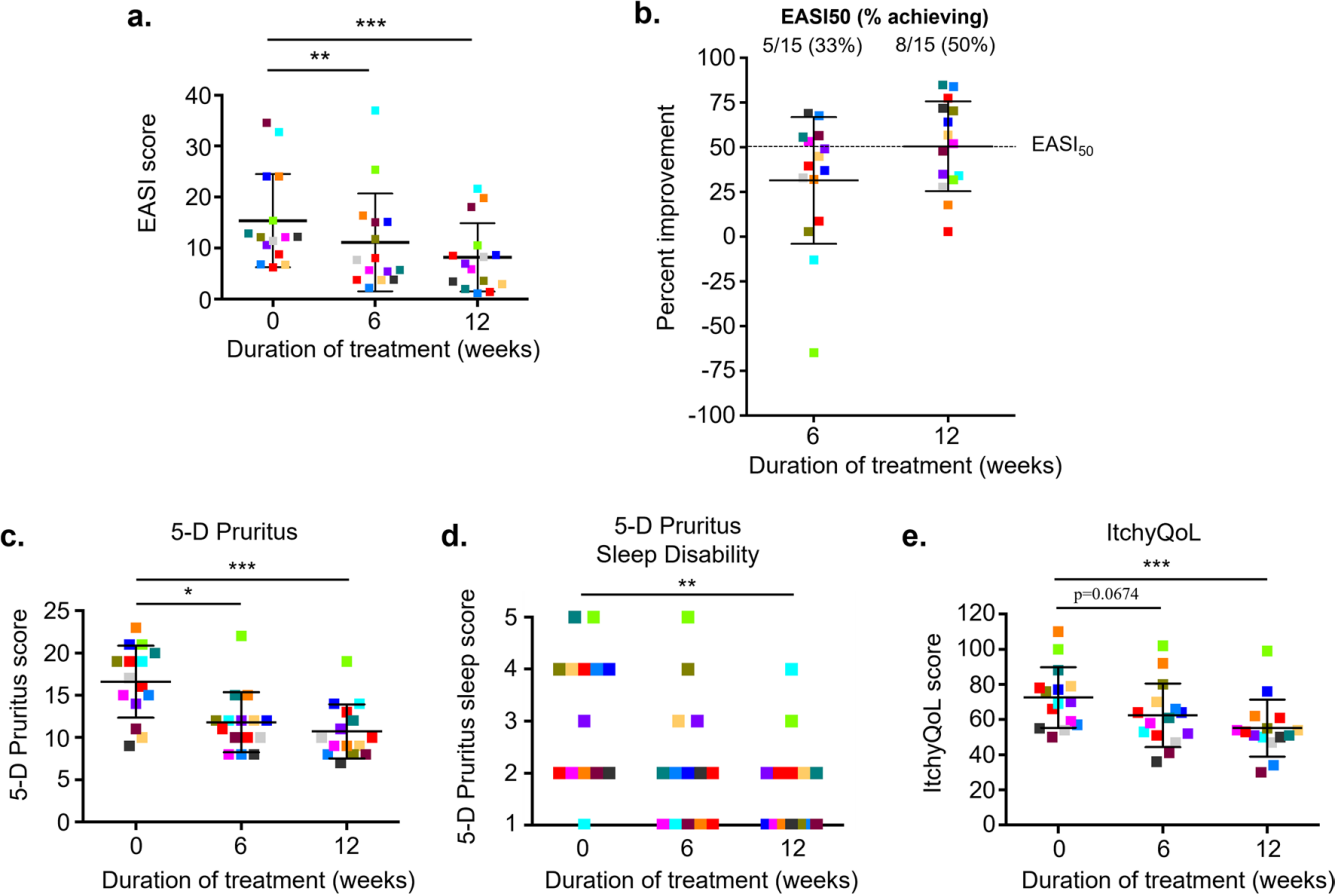
Bleach bath treatment reduces atopic dermatitis disease severity and itch. (**a**) The absolute EASI of AD subjects before and after 6 and 12 weeks of bleach bath treatment are shown as well as the (**b**) percent improvement in EASI. The dotted line represents a 50% improvement, or EASI_50_. For (**a**) data are mean ± SD, *n* = 15 AD subjects, **P = 0.0057, ***P = 0.0002 by One-way ANOVA with Dunn’s multiple comparisons test. For (**b**) data are mean percent improvement of *n* = 15 AD subjects at 6 and 12 weeks. Colors represent the same subjects over time. Itch was quantified using the (**c**, **d**) 5-D Pruritus and (**e**) ItchyQoL™ questionnaires at the time of enrollment and after 6 and 12 weeks of bleach bath treatment. (**d**) The 5-D Pruritus sleep score values are: 1 = never affects sleep, 2 = occasionally delays falling asleep, 3 = frequently delays falling asleep, 4 = delays falling asleep and occasionally wakes me up at night, 5 = delays falling asleep and frequently wakes me up at night. Data are mean ± SD, *n* = 15 AD subjects. For (**c**) *P = 0.0105, ***P < 0.0001 (**d**) **P = 0.002 and (**e**) ***P < 0.0001 by One-way ANOVA with Dunn’s multiple comparisons test. Colors represent the same subjects over time

The population demographics and study flow diagram are summarized in Table 1 and Fig. SI available in the electronic supplementary material (ESM). A significant absolute reduction in EASI score was observed by 6 weeks (31.4% improvement) and was slightly greater after 12 weeks (50.5% improvement) of treatment (Fig. 1a). By 6 and 12 weeks, 33% (5/15) and 53% (8/15) of subjects, respectively, reached a reduction of at least 50% in the EASI score (EASI_50_) (Fig. 1b). The majority (75%) of the severe AD subjects surpassed the minimal clinically important difference (MCID), defined as a reduction of ≥ 6.6 points from baseline EASI [28], after 6 weeks of bleach baths, while a smaller percentage (25%) of subjects with moderate disease at baseline achieved an MCID at 6 weeks (ESM Table SI). Although serum total immunoglobulin E (IgE) and Type 2 chemokine biomarkers PARC/CCL18 and TARC/CCL17 are considered good predictors of AD severity [29, 30], they were not predictive of bleach bath efficacy in our study cohort (ESM Fig. S2).

**Table 1:** Baseline demographics and clinical characteristics by disease category

Demographics	AD	NA
*N*	15	5
Males—*n* (%)	9 (60%)	3 (60%)
Age—years (Mean ± SD)	43 ± 15	42 ± 9
RACE—*n* (%)		
Caucasian	8 (53%)	3 (60%)
African American	5 (33%)	1 (20%)
Other/Mix	2 (13%)	1 (20%)
Non-Hispanic	15 (100%)	4 (80%)
Hispanic	0	1 (20%)
Clinical Characteristics on entry (Mean ± SD)		
EASI	15.4 ± 9.2	ND
ItchyQoL™	72.5 ± 17.2	ND
5D-Pruritus	16.6 ± 4.3	ND
TEWL (g/h/m^2^)^a^	14.5 ± 5.1	8.9 ± 2.9
SC Integrity (AUC)^a^	108.1 ± 90.9	46.01 ± 16.8
Skin pH	5.5 ± 0.5	5.3 ± 0.5
SC hydration (Corneometer units)	31.0 ± 8.4	28.8 ± 5.9
IgE (kU/L)^a^	2334.7 ± 2661.3	231.5 ± 234.6
PARC/CCL18 (pg/ml)	6.7E4 ± 3.2E4	3.8E4 ± 1.2E4
TARC/CCL17 (pg/ml)^a^	4584.8 ± 7978.2	916.8 ± 405.2

*ND* not determined

^a^Statistically significant differences between AD and NA at time of entry

### Bleach baths reduce itch

AD subjects rated their itch at each visit by answering both the 5-D Pruritus and ItchyQoL™ questionnaires. The mean 5-D pruritus score was significantly reduced after 6 and 12 weeks of bleach baths (Fig. 1c). Sleep was ranked as one of the highest disabilities (5-D Pruritus subscore) in 12/15 subjects (80%) and this measure was significantly improved by 12 weeks in 87% of subjects (13/15) (Fig. 1d). Similarly, the ItchyQoL™ questionnaire revealed a clear trend of itch reduction after 6 weeks with 80% of subjects (12/15) noting decreased itch (*P* = 0.06) which became significant after 12 weeks of bleach baths, with 100% of subjects noting improvements in itch (Fig. 1e).

### Bleach baths improve skin barrier function

**Fig. 2:**
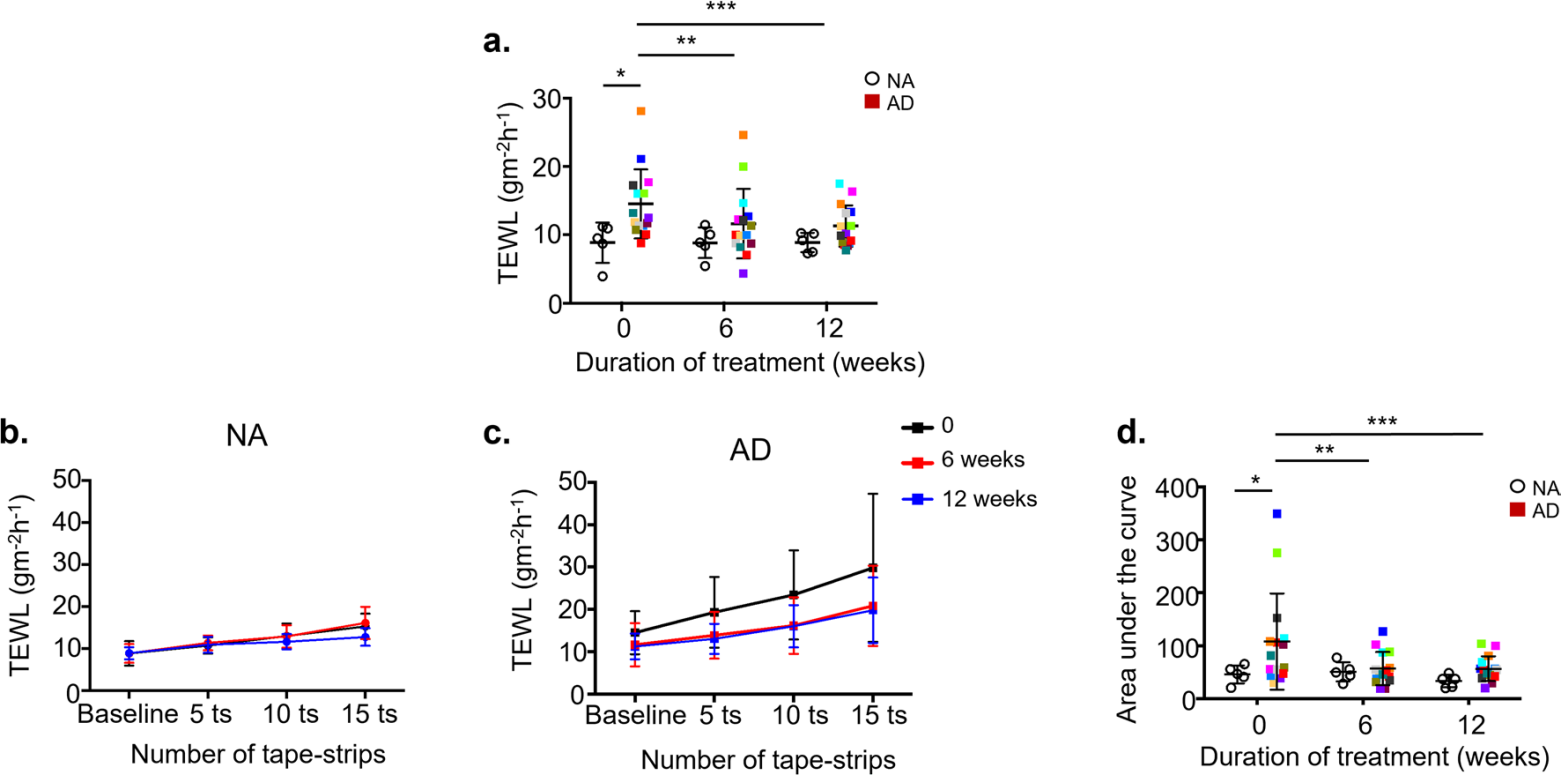
Bleach bath treatment improves skin barrier function. (**a**) TEWL in NA and AD subjects at baseline (pre-tape-stripping). (**b**) TEWL in NA and (**c**) AD at baseline and after 5, 10, and 15 tape-strips before treatment (black), after 6 weeks (red) and 12 weeks (blue) of bleach baths. (**d**) Normalized Area under the curve (AUC) calculations for TEWL values. Data are mean ± SD, *n* = 15 AD subjects and n = 5 NA subjects. For (**a**) *P = 0.0298 by Two-way ANOVA with Sidak’s multiple comparisons test, **P = 0.0121, ***P = 0.0043 by Two-way ANOVA with Tukey’s multiple comparisons test. For (**d**) *P = 0.01 by Two-way ANOVA with Sidak’s multiple comparisons test, **P = 0.0059, ***P = 0.0055 by Two-way ANOVA with Tukey’s multiple comparisons test. Colors represent the same subjects over time

Baseline TEWL values were significantly higher in AD subjects’ non-lesional skin compared to NA on study entry (14.5 ± 5.1 vs. 8.9 ± 2.9; *P* = 0.0298; Fig. 2a), indicating greater barrier dysfunction [31]. Baseline TEWL values significantly decreased in AD subjects but did not change in NA subjects with bleach bath treatment (Fig. 2a). Previous studies have suggested that AD skin has reduced SC hydration and higher pH compared to non-atopic skin [32]. However, we found no significant difference in these measures between AD and NA subjects on study entry and, importantly, no change following bleach baths (ESM Fig. S3a, b).

### Bleach baths significantly improve SC integrity in AD subjects

We evaluated SC integrity in non-lesional skin by measuring TEWL after 5, 10 and 15 sequential tape-strippings and reported the observation as an area under the curve (AUC) [33]. In a compromised skin barrier characteristic of AD, the AUC of the sequential TEWL measurements is greater than what is observed in healthy subjects [34]. In NA subjects, SC integrity was not altered after 6 or 12 weeks of bleach baths (Fig. 2b). AD subjects, however, experienced much more significant increases in TEWL after tape-stripping on study entry (black line, Fig. 2c), indicative of an SC defect [35, 36]. Bleach baths significantly improved SC integrity in AD subjects after 6 and 12 weeks (Fig. 2c). When the data is normalized to account for differences in baseline values (pre-tape-stripping) between NA and AD subjects, a significant difference between NA and AD AUC values at study entry is noted (Fig. 2d). However, only AD subjects had a significantly decreased AUC after 6 and 12 weeks of treatment (Fig. 2d).

### *S. aureus* growth and abundance is not altered by bleach baths

**Fig. 3:**
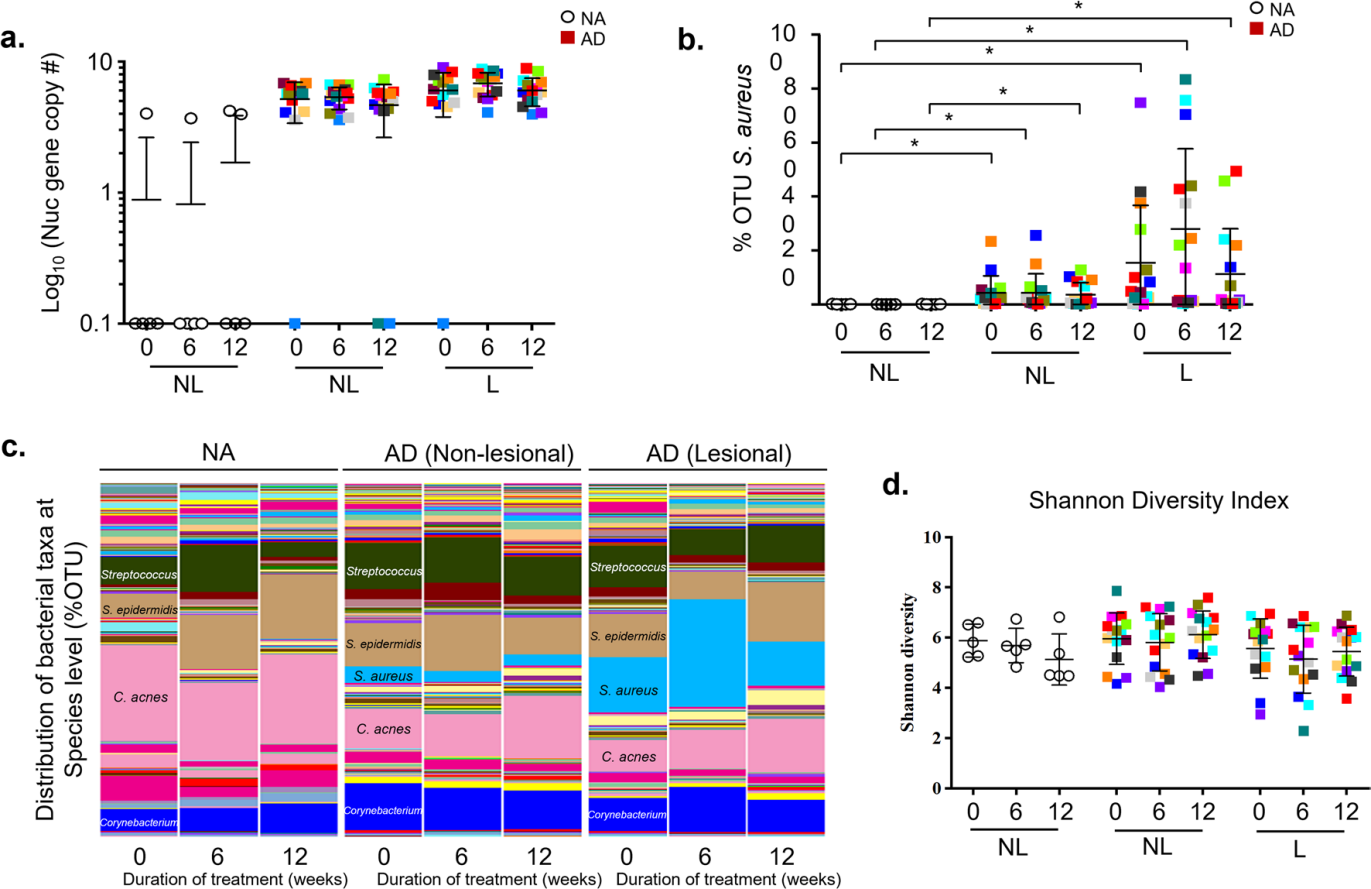
Bleach baths have no significant effect on *S. aureus* abundance or the skin microbiome. (**a**) *S. aureus* abundance (Log_10_ nuc gene copy number by qPCR) and (**b**) relative abundance (% operational taxonomic units [OTU] by high-throughput sequencing) were measured from skin swabs of normal skin of NA and non-lesional and lesional skin of AD taken before and after 6 and 12 weeks of bleach baths. (**c**) Bacterial taxonomic classifications at the species level from NA and AD non-lesional and AD lesional skin swabs before and after taking 6 and 12 weeks of bleach baths. (**d**) Shannon diversity analyses were performed using QIIME

Bleach baths did not reduce *S. aureus* on the skin, and the majority of AD subjects remained *S. aureus*-culture positive at non-lesional, lesional, or both skin sites after 12 weeks of bleach baths (data not shown). All subjects in the study were negative for methicillin-resistant S. aureus (MRSA). We performed qPCR and quantified the copy number of the thermonuclease (nuc) gene, which is specific for *S. aureus* and is a measure of the absolute abundance of both replicating and nonreplicating *S. aureus*. No significant changes were found in *S. aureus* abundance on non-lesional or lesional skin in NA or AD subjects following bleach baths (Fig. 3a).

### Bleach baths do not alter skin microbial diversity

We performed high-throughput sequencing of the variable regions, V1-V3, of the conserved 16S rRNA gene to obtain in-depth profiling of the skin microbiome on non-lesional (NA and AD) and lesional (AD) skin before and after bleach baths. The relative abundance of major genera and species is shown in ESM Fig. S4 and Fig. 3c. The mean ± SD of the distribution of the top 10 bacterial taxa at the species level is shown in ESM Table SII. We observed a significantly higher relative abundance [% Operational Taxonomic Units (OTU)] of *S. aureus* in AD non-lesional and lesional skin compared to NA skin at all time points (Fig. 3b, c). However, the % OTU of *S. aureus* was not significantly altered on AD non-lesional and lesional skin after taking bleach baths (Fig. 3b, c).

We also assessed whether bleach baths affected the microbial dysbiosis associated with AD skin by evaluating several measures of alpha diversity. The Shannon Diversity Index was unchanged at both non-lesional and lesional sites (Fig. 3d). The significant decreases in absolute EASI scores and itch did not significantly correlate with the Shannon Diversity Index, suggesting that improvements may not be driven by the relative changes in skin microbial populations (data not shown). Bleach baths had no effect on other measures of phylogenetic diversity including Chao1, PD Whole Tree and Strong Dominance Index (ESM Fig. S5a–c). Collectively, these data show that 12 weeks of bleach bath treatments do not significantly alter *S. aureus* cultivability and absolute abundance, or alter the relative abundance of the major bacterial skin communities. Furthermore, this highlights that reductions in absolute or relative amounts of *S. aureus* from AD skin may not be necessary to improve AD severity and symptomatology.

## Discussion

This study demonstrated that the addition of twice-weekly bleach baths in an adult, *S. aureus*-positive AD population improves disease severity, reduces itch and and itch-driven QoL measures, and enhances skin barrier functions. Notably, these improvements were seen without any reductions in *S. aureus* skin colonization measured by highly quantitative methods, suggesting that bleach baths improve AD through another mechanism. Importantly, bleach baths had no effect on skin hydration or pH in AD and NA subjects, suggesting that the alkalinity of bleach baths did not dry out the skin or worsen the abnormally elevated skin pH characteristically observed in AD subjects.

Our observation that bleach baths significantly reduce disease severity is in accordance with many studies that demonstrated significant improvements in clinical disease after bleach bath treatment [17–22]. As 75% of our study subjects with severe disease reached the MCID after 6 and 12 weeks, this highlights the possibility that bleach baths may be more effective in subjects with more severe disease or simply reaffirms that it is easier to achieve an MCID with a higher baseline EASI score. Further, Bakaa et al. noted a 22% relative decrease in AD severity with bleach bath treatment, resulting in a more pronounced decrease in EASI score for subjects with high disease activity compared to subjects with lower disease activity at baseline [37]. In contrast, Hon et al. found that four-week, twice-weekly bleach baths were not more useful than taking water baths in improving AD signs and symptoms in children [27]. Reasons for the discrepancies may be greater non-adherence to bleach baths or robustness of patient-reported measures in this younger population, the concomitant administration of oral antibiotics, or simply real biological differences in response to bleach in young versus adult AD skin [27].

The dominant symptom experienced by AD subjects is pruritus [38] which leads to a significant reduction in QoL [39]. Although remarkably little is known about what controls the intense itch associated with AD, several factors have been linked to itch, including prostaglandins, serotonin, beta-endorphins, proteases, and certain cytokines [38, 40]. Fukuyama et al. stimulated the dorsal root ganglia from mice treated with topical hydrochlorous acid or betamethasone dipropioinate with both histaminergic and non-histaminergic stimuli and found significantly less sensory nerve activation compared to untreated mice, suggesting that hypochlorous acid may play a role in sensory nerve desensitization. This study also showed that hypochlorous acid exhibits broad anti-inflammatory effects via decreased phosphorylation of MAP kinases and IkB pathways in dendritic cells after LPS exposure. However, these effects were specific to hypochlorous acid (HOCl), not hypochlorite (OCl-) which is the predominant form in bleach [41]. Notably, it was also found that itch intensity correlated with measures of skin barrier dysfunction (e.g. increased TEWL and decreased SC hydration) [42]. Consistent with this, we observed significant correlations between baseline TEWL and 5-D Pruritus and ItchyQoL™ scores (ESM Fig. S6a, b) in our study. Whether this suggests that the itch/scratch cycle is a key determinant of the skin barrier defect or that a disrupted barrier induces itch has yet to be determined. We also observed a strong correlation between baseline TEWL and EASI (ESM Fig. S6c), suggesting that bleach baths may improve disease severity by reducing itch and enhancing skin barrier function. Wong et al. found a significant reduction in itch by visual analog scale in AD subjects after two months of treatment with bleach baths, however, this improvement was no greater than that of the water bath control group [19].

SC dysfunction in AD is partially due to alterations in lipid composition/conformation [43, 44], reduced expression of structural proteins [45, 46], and simply the mechanical trauma of scratching [38]. Our data demonstrates that bleach baths improve barrier function, as seen by basal TEWL and SC integrity measurements (Fig. 2), which is in contrast to previously reported effects of bleach baths on barrier function [26, 27]. Our results indicate that the skin is less “leaky” (Fig. 2a) and suggest that bleach enhances corneocyte adhesion and/or improves the composition or organization of lipid lamellae. A previous study has shown that exposure to 0.05% diluted hypochlorite did not change ceramide or non-ceramide fractions of skin lipids in canine epidermal cell culture [47], which may suggest that positive effects of bleach baths on skin barrier are independent of skin lipid abnormalities common in AD. Future studies are needed to address the mechanism responsible for skin barrier enhancement and should include assessment of the skin transcriptome and lipidome.

Our results are consistent with other studies that noted improvements in AD with no significant reduction in *S. aureus* colonization after bleach baths [17, 19, 22]. Two of the studies used the same concentration and regimen as we used, while one used a 0.006% NaClO body wash for 6 weeks of daily use. Despite no differences in *S. aureus* colonization, Wong et al. observed a reduction in *S. aureus* density of 41.9% after 1-month and 53.3% after 3-months in children and young adults [19]. Huang et al. observed no significant reduction in *S. aureus* colonization at 1 month and 3 months despite 10 days of cephalexin therapy for baseline infection and monthly intranasal mupirocin treatment in conjunction with bleach baths [17]. Others have found recolonization within four weeks of stopping aggressive oral and topical antimicrobial treatments [7], suggesting the possibility of recolonization from body reservoirs such as the nares, perineum, or pharynx [48], or from close contact with partners and even household pets [49].

Gonzalez et al. were the first to analyze the skin microbiome of non-lesional and lesional skin in young children with and without AD and compare changes after treatment with topical corticosteroids alone or in conjunction with bleach baths [18]. Both treatments normalized lesional AD skin to more closely resemble non-lesional AD skin by the restoration of microbial diversity, decreased total bacterial burden, and decreased density of *Staphylococcus* species; however, both were still distinctly different than controls. Since their study did not include a group that took only bleach baths, we cannot know the relative contribution of bleach baths versus corticosteroids to the observed effects.

Our observation that bleach baths did not significantly alter the AD skin microbiome, despite improving severity, was unexpected. Since our AD subjects were required to be *S. aureus*-positive to be enrolled in our study, it was not surprising that we could detect *S. aureus* by culture, qPCR, and sequencing. Prior studies have shown selective shifts in the relative abundance of *S. aureus* during AD flares, with *S. aureus* reaching up to 90% of the total bacterial population [2]. We observed a more modest shift in the *Staphylococcus* genus or *S. aureus* species when comparing AD lesional with non-lesional skin or NA skin (Fig. 3c). We observed a high degree of variability between subjects, as noted by the large standard deviations, consistent with the published literature (ESM Table SII) [50]. Although we found no difference in *S. aureus* abundance, it is possible that bleach baths changed or reduced *S. aureus* toxins or virulence factors, which were not measured in our study.

In summary, our study confirms that bleach baths are safe and well-tolerated, corroborates published work demonstrating the ability of bleach baths to improve AD severity and itch, and is the first to show significant improvements in patient-reported outcomes of itch-related QoL, namely sleep, after bleach bath treatment. Importantly, the itch benefit (and EASI) correlated with improvements in skin barrier function and not the quantity of *S. aureus* on the skin surface (ESM Fig. S6). Future studies are needed to understand the mechanism(s) responsible for this bleach bath-induced skin barrier and itch improvement and to determine whether changes precede improvements in the signs of the disease (EASI).

## Material and methods

### Study population

Adults 18–65 years (inclusive) with mild-severe AD were recruited. Inclusion criteria included: EASI score greater than 6.0, *S. aureus* culture positivity, access to a bathtub, and willingness to maintain a stable dose of topical or systemic treatments for the duration of the study. Fifteen AD and five NA subjects completed all visits and met compliance requirements. NA were defined as having no personal or first-degree relative with a history of atopy and were comparable to the AD group in age, gender, and race. All NA subjects were negative for *S. aureus* by standard culture techniques. The study protocol was approved by the University of Rochester Medical Center RSRB (NCT01996150).

### Study design

Study participants were each given a bottle of 8.25% concentrated Clorox® bleach with a measuring cup marked to 3-oz volume. Participants were instructed to dilute 3 oz of bleach in approximately 40 gallons of water (final concentration 0.005% NaClO in a standard bathtub) and bathe for 5–10 min, 2x/week for a total of 12 weeks. Subjects were evaluated at 3 different time points: baseline (before bleach baths), 6 and 12 weeks after bleach baths.

### Assessments

#### EASI

EASI was used to measure the extent and severity of AD. Mild disease is defined by EASI scores of 1.1–7.0, moderate by 7.1–21.0, severe as 21.1–50.0, and very severe 50.1–72 [51]. No subjects enrolled met the very severe category. The MCID for EASI is calculated by the difference between EASI scores.

#### Itch

Pruritus was quantified using two validated patient-reported questionnaires: 5-D pruritus scale (Scale 5–25) [52] and ItchyQoL™ (Scale 22–110) [53]. The 5-D pruritus scale scores itch in 5 domains: 1. duration (how many hours spent itching), 2. degree (itch intensity), 3. direction (itch improved or worsened compared to the previous month), 4. disability (quality of life), and 5. distribution (body part). ItchyQoL™ is a 22-item questionnaire that assesses the quality of life related to itch in three main domains: symptoms, functioning, and emotions.

#### Skin barrier measurements

TEWL (Aquaflux, Biox Systems Ltd, London, UK), pH, and skin hydration measurements (Courage & Khazaka, Cologne, Germany) were obtained from a non-lesional site on the non-sun-exposed volar forearm in both AD and NA subjects prior to and after repeated tape-strippings (5, 10 and 15 Cuderm™ tape applications) using the Cuderm™ pressure device [54, 55]. This site could not be treated with medications or moisturizers within the previous 24 h.

#### S. aureus culture

Skin swabs were collected to determine *S. aureus* colonization and methicillin resistance using standard culture methods in the CLIA-certified URMC Clinical Microbiology Laboratory. Non-lesional sites were selected immediately adjacent to sites on the volar (non-sun-exposed) forearm where skin barrier functions were performed. Lesional sites were selected from the same arm when possible. A 3 × 3 cm area of skin was swabbed using non-bacteriostatic saline moistened rayon-tipped BBL™ CultureSwabs™ (Becton, Dickinson and Company, Le Pont de Claix, France).

#### S. aureus abundance and microbiome

Skin swabs were collected as noted above and processed to extract total genomic DNA as previously described [56] using DNA extractions, MasterPure Yeast DNA Purification Kit (Epicentre, Madison, WI) and PureLink Genomic DNA Mini Kit (Invitrogen) (see ESM).

### Supplementary Information

Below is the link to the electronic supplementary material.

Supplementary file1 (PDF 1121 KB)

## Data Availability

The datasets generated during and/or analysed during the current study are available from the corresponding author upon reasonable request.
