# Supplementary material for: Bleach baths enhance skin barrier, reduce itch but do not normalize skin dysbiosis in atopic dermatitis
Source: Arch Dermatol Res. 2023 Sep 27;315(10):2883–92. doi: 10.1007/s00403-023-02723-1 (PMC10615920; doi:10.1007/s00403-023-02723-1)
Supplement: Supplementary file 1 — Supplementary file1 (PDF 1121 KB) [file 403_2023_2723_MOESM1_ESM.pdf]

Electronic Supplementary material

Article title: Bleach Baths Enhance Skin Barrier, Reduce Itch but do not Normalize Skin Dysbiosis in Atopic Dermatitis

Journal: *Archives of Dermatological Research*

Authors: Ania Stolarczyk BS, Nelissa Perez-Nazario PhD, RN, Sara A. Knowlden PhD, Ellen Chinchilli BA, Alex Grier MS, Amy Paller MD, Steven R. Gill PhD, Anna De Benedetto MD, Takeshi Yoshida PhD, Lisa A. Beck MD

Corresponding Author:
Lisa A. Beck, MD
University of Rochester Medical Center
Department of Dermatology
601 Elmwood Ave, Box 697
Rochester, NY 14642
Email: lisa_beck@urmc.rochester.edu
585-275-1039 (phone)

Fig. S1 Study design flow chart.

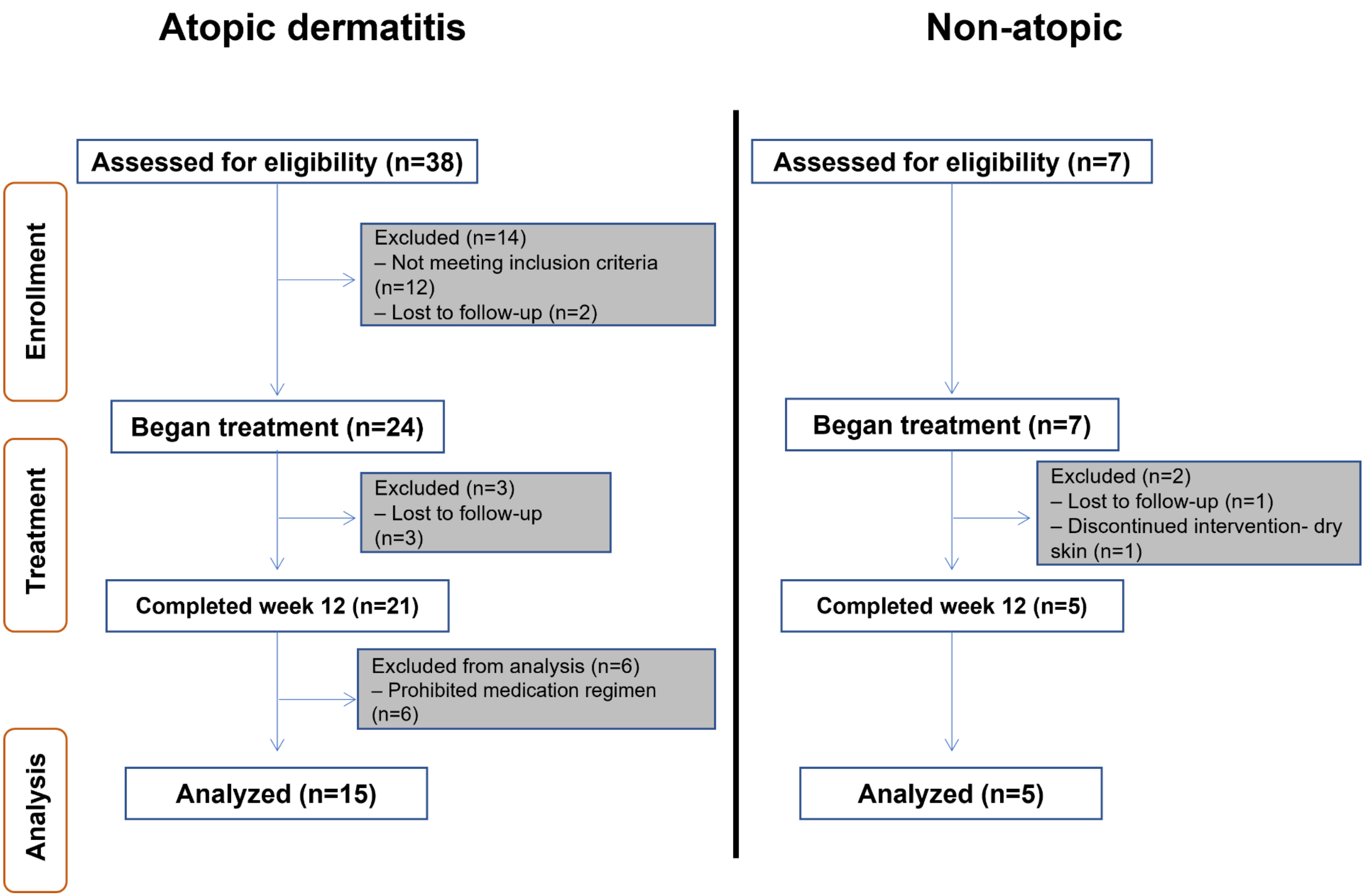

Of 38 AD subjects consented and screened, 24 met criteria to begin the study with 21 completing the study. Of the 21 subjects, 6 were excluded from data analysis due to medication regimen change during the study. No AD subjects withdrew from the study because of intolerance to the bleach baths and no adverse events were reported. One NA subject withdrew due to skin dryness that he thought was aggravated by the bleach baths.

Table SI. The number of AD subjects that reached a MCID[a] in EASI after 6 or 12 weeks of bleach bath treatment.

		**Duration of Bleach Baths**			
**Disease severity on entry**	**EASI on entry (Mean ± SD)**	**6 weeks**		**12 weeks**	
		**# of subjects (%)**	**Mean EASI change**	**# of subjects (%)**	**Mean EASI change**
Mild 1.1–7.0 (n=3)	6.6 ± 0.3	0/3 (0%)	-3.35	0/3 (0%)	-4.77
Moderate 7.1-21.0 (n=8)	11.9 ± 1.9	2/8 (25%)	-2.76	3/8 (37.5%)	-5.81
Severe 21.1-50.0 (n=4)	28.8 ± 5.6	3/4 (75%)	-7.96	3/4 (75%)	-11.81

[a]MCID = minimally clinically important difference for the EASI Severity measure is 6.6
(REF: *Allergy*. 2012 Jan;67(1):99-106.)

Fig. S2 Bleach baths have no effect on Type 2 serum biomarkers

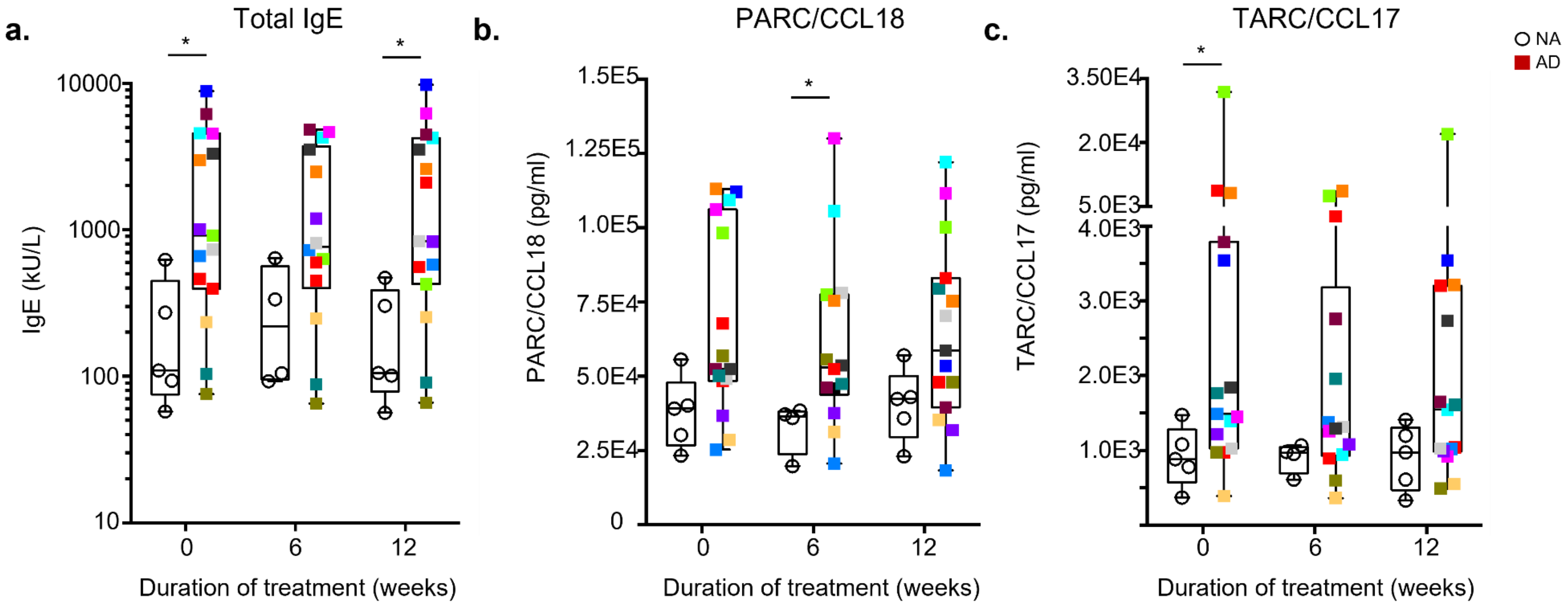

(a) Total IgE, (b) PARC/CCL18, and (c) TARC/CCL17 were measured in the serum of NA and AD subjects before and after 6 and 12 weeks of bleach baths. Data are shown as box and whisker plots. For (a) *P= 0.0193 (0 weeks), *P= 0.0193 (12 weeks); (b) *P= 0.0248 (6 weeks); (c) *P=0.0315 (0 weeks) by Mann-Whitney test. Colors represent the same subjects over time.

Fig. S3 Bleach bath treatment does not alter SC hydration or pH.

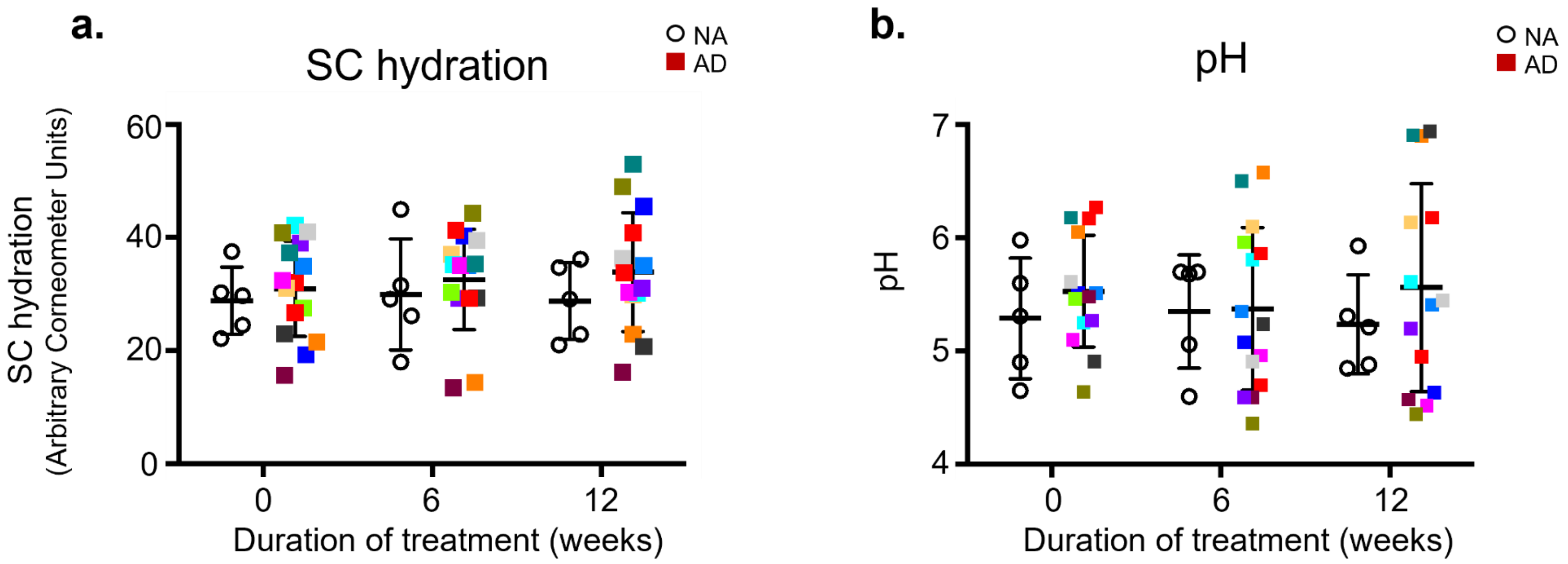

(a) SC hydration and (b) pH were measured in NA and AD subjects on non-lesional skin before bleach bath treatment and after 6 and 12 weeks of bleach bath treatments. There was no significant difference between NA and AD subjects at baseline. Colors represent the same subjects over time.

Fig. S4 Bacterial taxonomic classifications at the genus level from NA and AD non-lesional and lesional skin before and after taking 6 and 12 weeks of bleach baths.

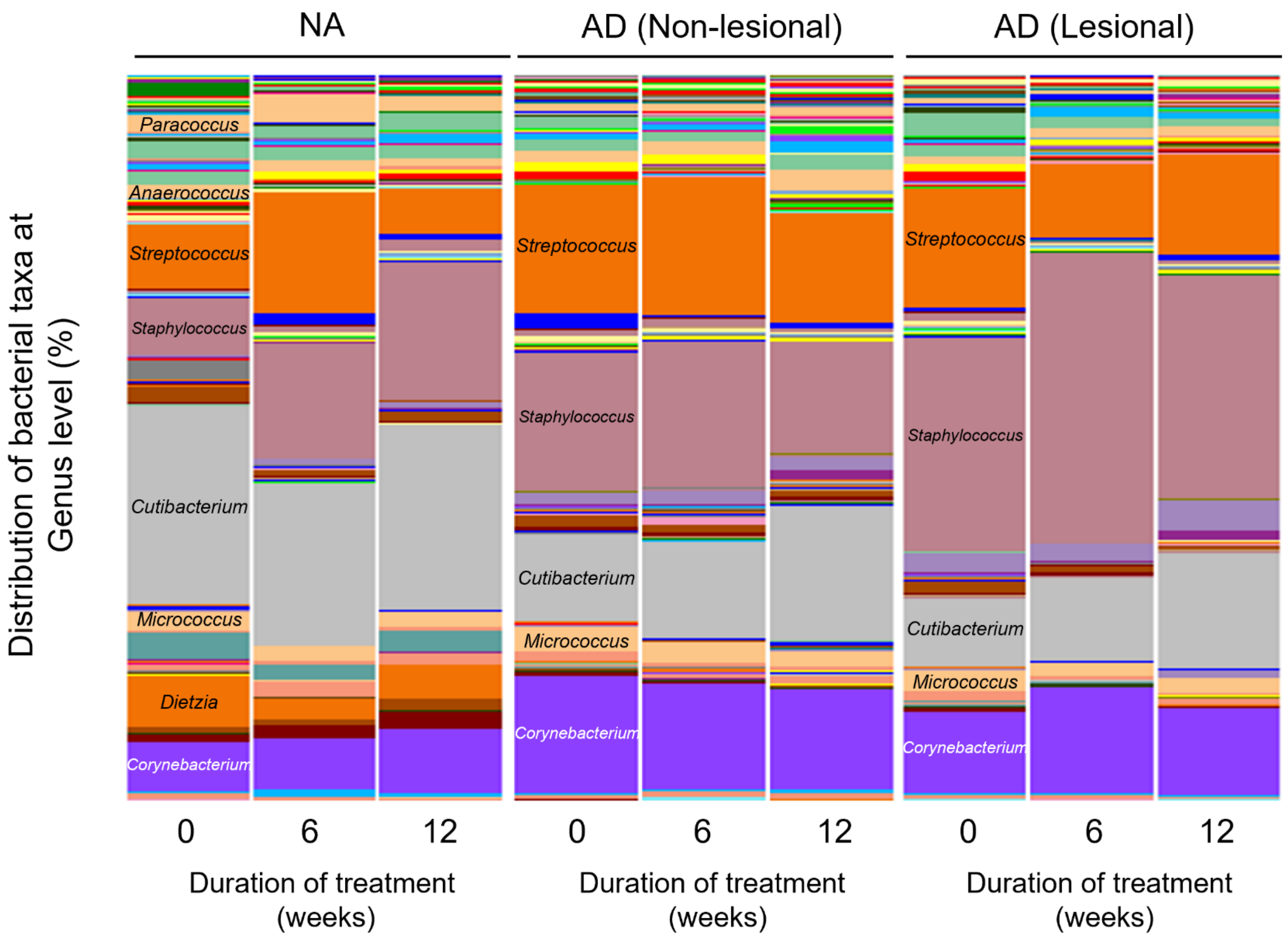

Table SII. Relative abundance of the top 10 most abundant bacterial taxa at the species level (Mean % OTUs[a] ± SD).

	NA (non-lesional)			AD (non-lesional)			AD (lesional)		
**Species**	**0**	**6**	**12**	**0**	**6**	**12**	**0**	**6**	**12**
P. acnes	26.4 ± 14.3%	24.3 ± 14.9%	25.3 ± 17.4%	12.4 ± 13.0%	13.7 ± 16.0%	15.0 ± 13.9%	10.0 ± 12.8%	12.0 ± 13.6%	14.7 ± 16.2%
S. epidermidis	5.9 ± 3.5%	13.0 ± 16.8%	13.28 ± 21.5%	12.6 ± 13.7%	13.0 ± 17.2%	10.8 ± 8.8%	13.4 ± 17.3%	9.3 ± 10.7%	17.4 ± 12.6%
*Corynebacterium;s_*	8.2 ± 4.3%	7.5 ± 5.9%	11.4 ± 11.2 %	14.4 ± 9.4%	12.4 ± 11.3%	11.9 ± 7.4%	11.2 ± 8.4%	12.6 ± 14.7%	9.3 ± 7.2%
*Streptococcus;s_*	6.4 ± 8.3%	12.5 ± 8.3%	4.2 ± 3.3%	11.3 ± 12.4%	12.4 ± 12.9%	10.9 ± 9.0%	10.4 ± 9.6%	6.8 ± 6.2%	10.3 ± 9.8%
S. aureus	0.1 ± 0.1	0.02 ± 0.04%	0.06 ± 0.1%	4.3 ± 6.3%	4.3 ± 7.1%	3.6 ± 4.6%	15.4 ± 21.4%	28.0 ± 29.7%	11.3 ± 16.8%
Streptococcus; Other	0.9 ± 0.8%	2.2 ± 0.9%	0.9 ± 0.7%	2.6 ± 5.3%	4.2 ± 8.0%	2.5 ± 2.8%	2.5 ± 4.1%	2.0 ± 2.8%	2.6 ± 5.1%
M. luteus	2.6 ± 2.0%	1.8 ± 1.4%	2.0 ± 1.1%	2.8 ± 3.5%	2.7 ± 4.4%	3.0 ± 4.7%	2.8 ± 5.1%	1.7 ± 3.2%	1.7 ± 3.6%
Bacilli; Other	0.5 ± 0.3%	0.5 ± 0.5%	0.7 ± 0.8%	1.7 ± 1.0%	1.5± 1.3%	1.8 ± 1.2%	2.8 ± 1.7%	2.5 ± 2.2%	4.3 ± 6.0%
*Anaerococcus;s_*	2.8 ± 2.6%	1.4 ± 1.0%	0.9 ± 0.7%	2.3 ± 4.1%	2.1 ± 4.8%	2.6 ± 5.5%	1.8 ± 3.4%	1.5 ± 3.1%	1.9 ± 4.3%
*Finegoldia;s_*	2.0 ± 2.0%	1.6 ± 1.6%	1.6 ± 1.1%	1.6 ± 2.0%	1.3 ± 2.1%	2.0 ± 2.8%	1.6 ± 2.3%	1.6 ± 2.8%	1.1 ± 1.3%

[a] OTU=operational taxonomic unit

Fig. S5 Bleach baths do not change alpha diversity on NA or AD skin.

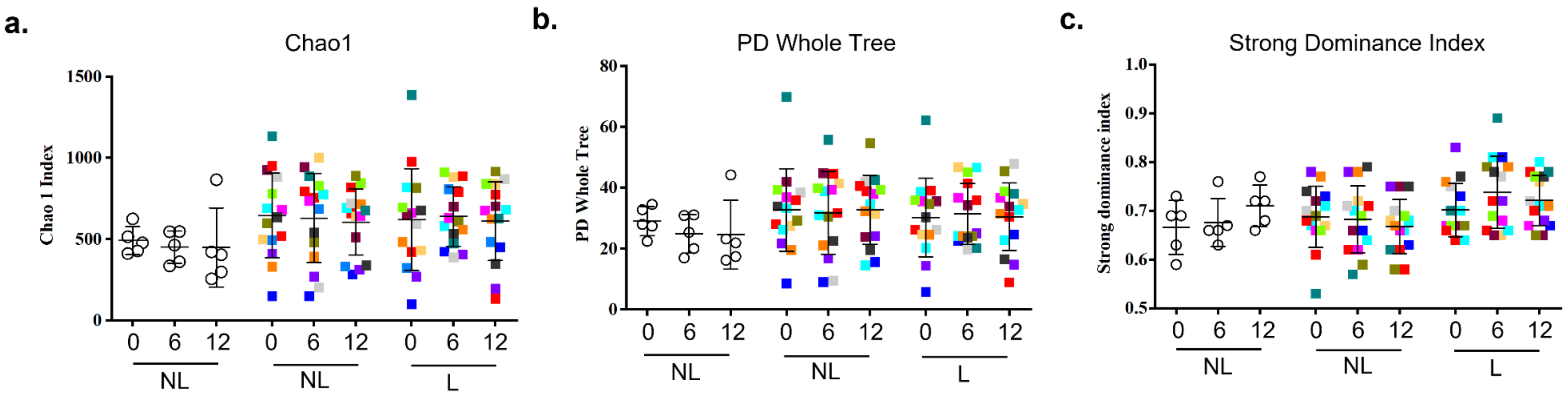

(a) Chao1, (b) PD Whole Tree, and (c) Strong Dominance Index analyses were performed using QIIME, with a non-parametric t-test corrected for multiple testing to assess differences between groups.

Fig. S6 A correlation was observed between TEWL measurements taken at any time point in the study from AD non-lesional skin and (a) 5-D Pruritus, (b) ItchyQoL™, and (c) EASI.

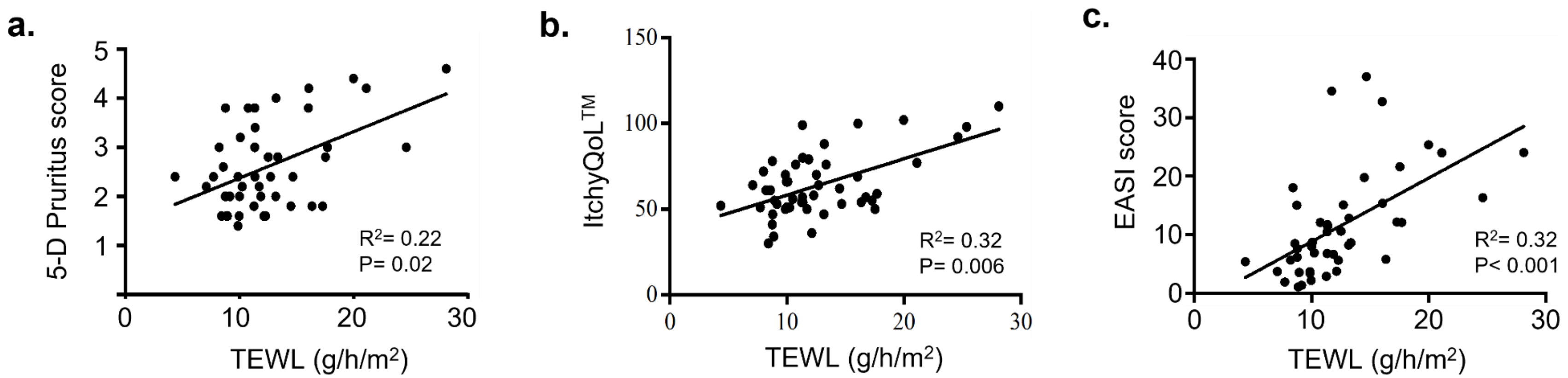

Statistical analyses for R^2 found by linear regression and P-values by correlations.

Supplemental Materials and Methods

***S. aureus abundance and microbiome*:** Samples processed to extract total genomic DNA as previously described [1] using DNA extractions, MasterPure Yeast DNA Purification Kit (Epicentre, Madison, WI) and PureLink Genomic DNA Mini Kit (Invitrogen) with the following modifications: the Catch-All Sample Collection Swabs (Epicentre, Madison, WI) were pre-moistened with sterile saline and rubbed on the skin up and down 10 times, back and forth 10 times, and rolled 10 times, and stored in 100 μl of yeast cell lysis buffer (MasterPure Yeast DNA Purification Kit, Epicentre) at -80°C. Mechanical disruption was performed using 0.1 mm silica spheres (MP Biomedicals, Santa Ana, CA) in the Bead Ruptor$_{12}$ (Omni International, NW Kennesaw, GA). *S. aureus* abundance was measured by SYBR green qPCR (Biorad, Hercules, CA) using specific primers for the thermonuclease (nuc) gene. 16S ribosomal RNA (rRNA) was amplified with Q5 DNA polymerase (New England Biolabs, Ipswich, MA) and dual indexed V1-V3 primers [2]. Amplicons were pooled and paired-end sequenced (2 X 300 nt) on an Illumina MiSeq (Illumina, San Diego, CA) in the University of Rochester Genomics Research Center. Each sequencing run included: (1) positive controls consisting of a 1:5 mixture of *S. aureus*, *Lactococcus lactis*, *Porphyromonas gingivalis*, *Streptococcus mutans*, and *Escherichia coli*; and (2) negative controls consisting of sterile saline.

Sequence Data Processing: Raw data from the Illumina MiSeq was converted into FASTQ format 2x300 paired end sequence files using the bcl2fastq program, version 1.8.4, provided by Illumina. Format conversion was performed without de-multiplexing and the EAMMS algorithm was disabled. All other settings were default. Sequence processing and microbial composition analysis were performed with the Quantitative Insights into Microbial Ecology (QIIME) software package [3], version 1.9. Reads were multiplexed using a configuration described previously [2]. Briefly, for both reads in a pair, the first 12 bases were a barcode, which was followed by a primer, then a heterogeneity spacer, and then the target 16S rRNA sequence. Using a custom Python script, the barcodes from each read pair were removed, concatenated together, and stored in a separate file. Read pairs were assembled using fastq-join from the ea-utils package, requiring at least 20 bases of overlap and allowing a maximum of 10% mismatched bases. Read pairs that could not be assembled were discarded. The concatenated barcode sequences were prepended to the corresponding assembled reads, and the resulting sequences were converted from FASTQ to FASTA and QUAL files for QIIME analysis. Barcodes, forward primer, spacer, and reverse primer sequences were removed during de-multiplexing. Reads containing more than four mismatches to the known primer sequences or more than three mismatches to all barcode sequences were excluded from subsequent processing and analysis. Assembled reads were truncated at the beginning of the first 30 base window with a mean Phred quality score of less than 20 or at the first ambiguous base, whichever came first. Resulting sequences shorter than 300 bases or containing a homopolymer longer than six bases were discarded. Operational taxonomic units (OTU) were picked using the reference-based USEARCH (version 5.2) [4] pipeline in QIIME, using the May 2013 release of the GreenGenes 99% OTU database as a closed reference [5, 6]. An indexed word length of 128 and otherwise default parameters were used with USEARCH. Chimera detection was performed *de novo* with UCHIME,

using default parameters [4]. OTU clusters with less than four sequences were removed, and representative sequences used to make taxonomic assignments for each cluster were selected on the basis of abundance. The RDP Naïve Bayesian Classifier was used for taxonomic classification with the GreenGenes reference database, using a minimum confidence threshold of 0.85 and otherwise default parameters [7].

1. Oh J, Byrd AL, Deming C, Conlan S, Program NCS, Kong HH, et al. Biogeography and individuality shape function in the human skin metagenome. Nature. 2014;514(7520):59-64. DOI: 10.1038/nature13786.
2. Fadrosh DW, Ma B, Gajer P, Sengamalay N, Ott S, Brotman RM, et al. An improved dual-indexing approach for multiplexed 16S rRNA gene sequencing on the Illumina MiSeq platform. Microbiome. 2014;2(1):6. DOI: 10.1186/2049-2618-2-6.
3. Caporaso JG, Kuczynski J, Stombaugh J, Bittinger K, Bushman FD, Costello EK, et al. QIIME allows analysis of high-throughput community sequencing data. Nature methods. 2010;7(5):335-6. DOI: 10.1038/nmeth.f.303.
4. Edgar RC, Haas BJ, Clemente JC, Quince C, Knight R. UCHIME improves sensitivity and speed of chimera detection. Bioinformatics. 2011;27(16):2194-200. DOI: 10.1093/bioinformatics/btr381.
5. DeSantis TZ, Hugenholtz P, Larsen N, Rojas M, Brodie EL, Keller K, et al. Greengenes, a chimera-checked 16S rRNA gene database and workbench compatible with ARB. Appl Environ Microbiol. 2006;72(7):5069-72. DOI: 10.1128/AEM.03006-05.
6. McDonald D, Price MN, Goodrich J, Nawrocki EP, DeSantis TZ, Probst A, et al. An improved Greengenes taxonomy with explicit ranks for ecological and evolutionary analyses of bacteria and archaea. ISME J. 2012;6(3):610-8. DOI: 10.1038/ismej.2011.139.
7. Wang Q, Garrity GM, Tiedje JM, Cole JR. Naive Bayesian classifier for rapid assignment of rRNA sequences into the new bacterial taxonomy. Appl Environ Microbiol. 2007;73(16):5261-7. DOI: 10.1128/AEM.00062-07.
